# Metabolism of 25-Hydroxy-Vitamin D in Human Macrophages Is Highly Dependent on Macrophage Polarization

**DOI:** 10.3390/ijms231810943

**Published:** 2022-09-19

**Authors:** Rie H. Nygaard, Marlene C. Nielsen, Kristian W. Antonsen, Carsten S. Højskov, Boe S. Sørensen, Holger J. Møller

**Affiliations:** 1 Department of Clinical Biochemistry, Aarhus University Hospital, 8200 Aarhus, Denmark; 2 Department of Clinical Medicine, Aarhus University, 8200 Aarhus, Denmark

**Keywords:** inflammation, immune system, monocyte-derived macrophages, 3-epi-25-hydroxy-vitamin D, qPCR, liquid chromatography-mass spectrometry

## Abstract

Macrophages synthesize active vitamin D (1,25-dihydroxy-vitamin D) and express the vitamin D receptor in the nucleus; however, vitamin D metabolism in relation to macrophage polarization and function is not well understood. We studied monocyte-derived macrophages (MDMs) from human buffy coats polarized into M0, M1 (LPS + IFNγ), M2a (IL4 + IL13) and M2c (IL10) macrophage subtypes stimulated with 25-hydroxy-vitamin D (1000 and 10,000 nanomolar). We measured vitamin D metabolites (25-hydroxy-vitamin D, 1,25-dihydroxy-vitamin D, 24,25-dihydroxy-vitamin D and 3-epi-25-hydroxy-vitamin D) in cell media with liquid chromatography-mass spectrometry-mass spectrometry. The mRNA expression (*CYP27B1*, *CYP24A1* and *CYP24A1-SV*) was measured with qPCR. We found that reparative MDMs (M2a) had significantly more 1,25-dihydroxy-vitamin D compared to the other MDMs (M0, M1 and M2c). All MDMs were able to produce 3-epi-25-hydroxy-vitamin D, but this pathway was almost completely attenuated in inflammatory M1 MDMs. All MDM subtypes degraded vitamin D through the 24-hydroxylase pathway, although M1 MDMs mainly expressed an inactive splice variant of *CYP24A1,* coding the degrading enzyme. In conclusion, this study shows that vitamin D metabolism is highly dependent on macrophage polarization and that the C3-epimerase pathway for vitamin D is active in macrophages.

## 1. Introduction

It has been known for several years that vitamin D has other functions besides the regulation of calcium and bone homeostasis. One of these important functions is the role of vitamin D in regulating the immune system. Overall, vitamin D is believed to have an anti-inflammatory effect, but its actions are complex [1,2]. Whereas the majority of human cells, including macrophages, have the vitamin D receptor (VDR) in their nucleus, *CYP27B1* activity and the production of active vitamin D (1,25-dihydroxy vitamin D) outside the kidney are restricted to macrophages and a few other cell types [2,3]. This extra-renal production of active vitamin D is believed to be crucial for many of the local effects of vitamin D, allowing autocrine and paracrine signaling with high levels of active vitamin D in target tissues.

1,25-dihydroxy-vitamin D is also known to affect macrophage polarization and induce switching from inflammatory macrophages (M1 macrophages) toward reparative macrophages (M2 macrophages) [4,5]. This polarization is believed to be a continuum and the cells have a great plasticity and are able to polarize according to the microenvironment [6]. Regulation of local vitamin D activity also relies on the degradation of 1,25-dihydroxy-vitamin D and its precursor 25-hydroxy-vitamin D. The main degrading pathway through the 24-hydroxylase (CYP24A1) has been proposed to be compromised in macrophages, since human macrophages have been suggested to partly express a nonfunctional splice variant of the degrading enzyme (CYP24A1-SV) [7]. A less well understood metabolic pathway of vitamin D is the C3-epimerase pathway, where an unknown enzyme (C3-epimerase) alters the orientation of the hydroxy-group on the carbon in the third position of the vitamin D molecule [8]. Cell studies have shown that the C3-epimerase is active in human hepatocytes, keratinocytes, osteoblasts, intestine cells and kidney cells [9,10,11]. The function of this pathway is unknown.

Since the local concentration of the active 1,25-dihydroxy-vitamin D is important for macrophage function and polarization, and since it has been suggested that the main degrading pathway of 25-hydroxy-vitamin D and 1,25-dihydroxy-vitamin D, by the 24-hydroxylase, is altered in macrophages, we found it important to investigate if macrophage polarization affected vitamin D metabolism and whether the C3-epimerase pathway is active in macrophages or not. We used the following human polarized monocyte-derived macrophages (MDMs): undifferentiated (M0), inflammatory (M1, stimulated with LPS + IFNγ), reparative (M2a, stimulated with IL4 + IL13) and deactivated (M2c, stimulated with IL10). We studied the effect of 25-hydroxy-vitamin D on secreted vitamin D metabolites and mRNA expression of the known enzymes for synthesis of 1,25-dihydroxy-vitamin D (CYP27B1) and for 24,25-dihydroxy-vitamin D (CYP24A1).

## 2. Results

### 2.1. Baseline and Cell Phenotype

**Figure 1:**
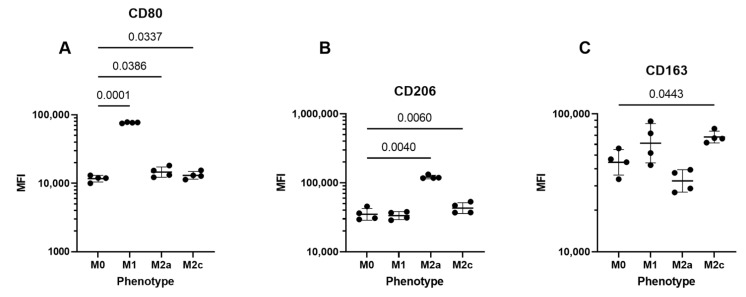
Flow cytometry measures of MDM surface markers. MDMs were polarized into M0, M1, M2a and M2c. Cells were harvested and the surface markers CD80 (**A**), CD206 (**B**) and CD163 (**C**) were measured with flow cytometry. The numbers above the horizontal lines indicate the different *p*-values.

We found no detectable vitamin D metabolites (25-hydroxy-vitamin D, 3-epi-25-hydroxy-vitamin D, 24,25-dihydroxy-vitamin D or 1,25-dihydroxy-vitamin D) in any of the cell media not stimulated with 25-hydroxy-vitamin D (LCMSMS analysis on cell media, n = 10). Furthermore, we analyzed cell media from all donors 5 min after stimulation with 25-hydroxy-vitamin D and found no detectable 3-epi-25-hydroxy-vitamin D, 24,25-dihydroxy-vitamin D or 1,25-dihydroxy-vitamin D (LCMSMS analysis on cell media, n = 10). Based on these results, we concluded that there were no significant vitamin D metabolites in the cell cultures or added stripped sera before stimulation with 25-hydroxy-vitamin D, and that the stimulation did not include any 3-epi-25-hydroxy-vitamin D. The polarization of the MDM cells was carried out using commonly used growth factors [6]. To evaluate whether this polarization protocol did indeed result in the expected cell phenotypes, we measured surface markers on separate cells. As expected, CD80 was upregulated in M1 MDMs, CD206 was upregulated in M2a MDMs, and CD163 was upregulated in M1 and M2c MDMs (Figure 1).

### 2.2. MDMs Are Able to Synthesize 3-Epi-25-Hydroxy-Vitamin D from 25-Hydroxy-Vitamin D

In Donor 1-4, we found that M0 MDMs synthesized and secreted 3-epi-25-hydroxy-vitamin D in response to stimulation with either 1000 nM or 10,000 nM 25-hydroxy-vitamin D in three out of the four donors (Table 1). The 3-epi-25-hydroxy-vitamin D metabolite was found in the media after 24 and 48 h of stimulation, and the concentration of 3-epi-25-hydroxy-vitamin D appeared linear to the substrate, with 1000 nM 25-hydroxy vitamin D leading to concentrations of 3-epi-25-hydroxy-vitamin D around 40 nM and 10,000 nM 25-hydroxy-vitamin D leading to 3-epi-25-hydroxy-vitamin D around 400–500 nM after 24 h. In three donors (donor 2–4), we also measured 3-epi-25-hydroxy-vitamin D in the media after 48 h. In media from two donors (donors 3 and 4), we observed increasing concentrations, while no 3-epi-25-hydroxy-vitamin D was measured in media from the third donor (donor 2).

### 2.3. Vitamin D Metabolism Is Dependent on MDM Polarization

**Figure 2:**
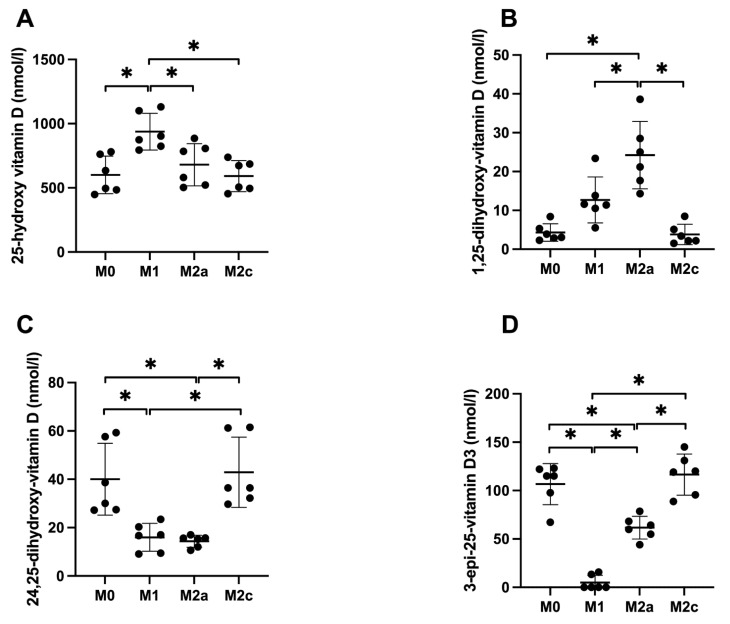
Vitamin D metabolites in polarized MDMs after vitamin D stimulation. MDMs were polarized into M0, M1, M2a and M2c MDMs and stimulated with 1000 nM 25-hydroxy-vitamin D. Concentrations of vitamin D metabolites in the different subtypes of MDMs were measured in the media after 24 h: 25-hydroxy-vitamin D (**A**), 1,25-dihydroxy-vitamin D (**B**), 24,25-dihydroxy-vitamin D (**C**) and 3-epi-25-hydroxy-vitamin D (**D**). * Indicates statistically significant differences between groups (subtypes) on each graph.

After showing that human M0 MDMs were able to produce 3-epi-25-hydroxy-vitamin D, we analyzed vitamin D metabolites in subtypes of MDMs (M0, M1, M2a and M2c) from Donor 5–10. All these cells were stimulated with 1000 nM 25-hydroxy-vitamin D for 24 h. These data are shown in Figure 2. The M1 MDMs showed little metabolism of the added 25-hydroxy-vitamin D, having almost the same level of 25-hydroxy-vitamin D in the media after 24 h (significantly higher than all 3 other MDM subtypes). The M1 MDMs secreted small amounts of 1,25-dihydroxy-vitamin D (significantly lower than M2a MDMs) and 24,25-dihydroxy-vitamin D (significantly lower than M0 and M2c MDMs), and almost no 3-epi-25-hydroxy-vitamin D (significantly lower than all 3 other MDM subtypes). In contrast, the M2a MDMs produced the highest amount of active 1,25-dihydroxy-vitamin D (significantly higher than all 3 other MDM subtypes) and showed relatively low concentrations of both 24,25-dihydroxy-vitamin D (significantly lower than M0 and M2c MDMs) and 3-epi-25-hydroxy-vitamin D (significantly lower than M0 and M2c MDMs). The M2c MDMs did not differ significantly from the M0 MDMs in the concentrations of any vitamin D metabolites.

### 2.4. The mRNA Expression of CYP27B1, CYP24A1 and CYP24A1-SV

**Figure 3:**
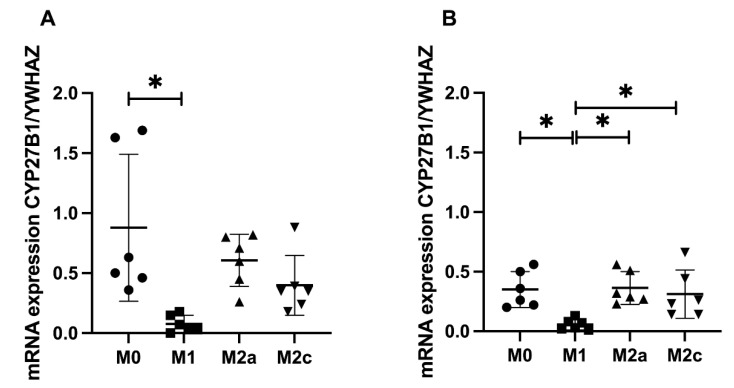
Expression of *CYP27B1* in polarized MDMs with and without vitamin D stimulation. MDMs were polarized into M0, M1, M2a and M2c MDMs and stimulated with either 0 nM (**A**) or 1000 nM (**B**) 25-hydroxy-vitamin D for 24 h. The mRNA expression of *CYP27B1* is shown relative to the household gene (*YWHAZ*). * Indicates a statistically significant difference between groups.

The mRNA expression data for *CYP27B1* are shown in Figure 3a,b. The CYP27B1 enzyme (1α-hydroxylase) is responsible for conversion of 25-hydroxy-vitamin D into 1,25-dihydroxy-vitamin D. *CYP27B1* mRNA was expressed in M0, M2a and M2c MDMs, both with and without the presence of its substrate (25-hydroxy-vitamin D). M1 MDMs had very low or no expression of *CYP27B1* mRNA, and in these cells, it was not induced by the presence of 25-hydroxy-vitamin D.

**Figure 4:**
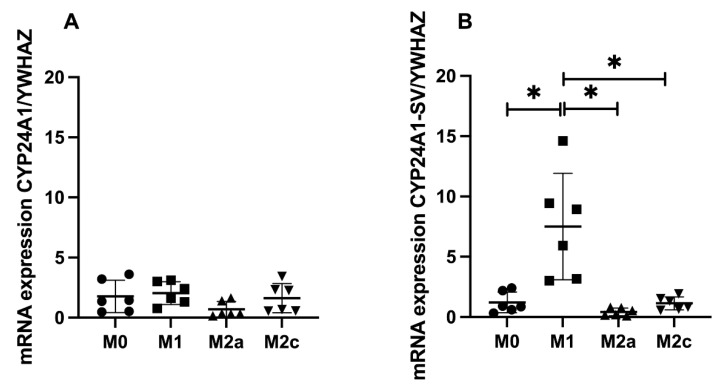
Expression of *CYP24A1* and *CYP24A1-SV* in polarized MDMs after vitamin D stimulation. MDMs were polarized into M0, M1, M2a and M2c MDMs and stimulated with 1000 nM 25-hydroxy-vitamin D for 24 h. The mRNA expression of *CYP24A1* is shown in (**A**) and the mRNA expression of its splice variant, *CYP24A1*, is shown in (**B**). Both expressions are shown relative to a household gene (*YWHAZ*). * Indicates a statistically significant difference between groups.

In contrast to *CYP27B1*, we found no mRNA expression of *CYP24A1* and *CYP24A1-SV* without stimulation of 25-hydroxy-vitamin D. The mRNA expression data after stimulation with 25-hydroxy-vitamin D of *CYP24A1* and *CYP24A1-SV* are shown in Figure 4a,b. The mRNA expression of *CYP24A1* in response to 25-hydroxy-vitamin D did not differ between subtypes of MDMs, but the expression of the splice variant (*CYP24A1-SV*) was strongly induced in M1 MDMs compared to the other three subtypes.

## 3. Discussion

The main finding of our study was that macrophage polarization clearly altered the cell response to vitamin D. The reparative M2a macrophages in our study secreted more 1,25-dihydroxy-vitamin D than both inflammatory cells (M1 MDMs), undifferentiated cells (M0 MDMs), and deactivated cells (M2c MDMs). Vitamin D degradation showed almost the opposite pattern, as the highest concentrations of 24,25-dihydroxy vitamin D were in the undifferentiated cells (M0 MDMs) and deactivated cells (M2c MDMs). The mRNA expression for the activating enzyme (CYP27B1) was continuously expressed with or without the vitamin D substrate present in all polarized cells except the inflammatory cells (M1). This was in contrast to the mRNA expression for the degrading enzyme (CYP24A1), which was only expressed after stimulation with vitamin D.

The inflammatory MDMs in our study (M1) responded very differently to vitamin D stimulus compared to the reparative M2a cells. They showed almost no mRNA expression of the enzyme converting 25-hydroxy-vitamin D to the active metabolite 1,25-dihydroxy-vitamin D (CYP27B1), either after stimulation with 25-hydroxy-vitamin D. Nevertheless, we found 1,25-dihydroxy-vitamin D in the media after stimulation with 25-hydroxy-vitamin D, but in lower concentrations compared to the reparative MDMs (M2a). A recent study found a similar increase in 1,25-dihydroxy-vitamin D production in M1 cells. However, in that study, the M1 cells showed a strong upregulation of the *CYP27B1* gene in response to 25-hydroxy-vitamin D, which is in contrast to the very low expression levels in our study [12]. We have no explanation for this difference. The “M2 cells”, in that study, were stimulated with LPS and not comparable to the high producing 1,25-dihydroxy-vitamin D M2a cells presented here.

We found the 24-hydroxylase pathway to be active in all subtypes of MDMs with secretion of 24,25-dihydroxy-vitamin D. We also confirmed that MDMs express a splice variant of *CYP24A1*, as described earlier, in macrophage-like cells (THP-1) [7]. Interestingly, we found that the splice variant of *CYP24A1* was especially upregulated in inflammatory MDMs (M1). This *CYP24A1-SV* has been suggested to be a potent attenuator of *CYP27B1* [7,13] which could explain the combination of high *CYP24A1-SV* expression and low *CYP27B1* expression in our M1 MDMs.

In human circulation, 3-epi-25-hydroxy vitamin D is found with very high inter-individual variation [14,15,16], and it has been shown that this metabolite of vitamin D can be synthesized in vivo [9,10,11]. To our knowledge, we are the first to show that human MDMs are able to synthesize 3-epi-25-hydroxy-vitamin D. Furthermore, this pathway was also found to be affected by MDM polarization, as it was almost absent in the inflammatory M1 MDMs. The implications of these findings are unclear since the function of this metabolic pathway is still unknown. However, it has been shown that the 3-epi-1,25-dihydroxy-vitamin D maintains some affinity for the vitamin D receptor [8], suggesting that the function of this pathway may not only be degradation [5].

Overall, our findings are in compliance with vitamin D’s known role as an anti-inflammatory agent in the immune system. Reparative macrophages will react to vitamin D, secrete the active form of vitamin D and continue anti-inflammatory signaling. However, our data suggest that vitamin D does not have the same effect on inflammatory macrophages. In vivo, macrophages are believed to be plastic, and a given tissue may harbor a continuum of polarized macrophages. The microenvironment of these cells may determine their properties, which can change over time [6]. Vitamin D also plays a role in determining this polarization, as several studies have shown that 1,25-dihydroxy-vitamin D can promote switching from M1 macrophages to M2 macrophages [4,17,18]. This switch from M1 macrophages to M2 macrophages is central to an inflammatory response. If the M1 response does not change over time to an M2 response, the inflammation will become chronic, and this mechanism is believed to contribute to the pathogenesis of many major inflammatory diseases, such as atherosclerosis, diabetes and inflammatory bowel disease [19,20]. Our data suggest that increased circulating 25-hydroxy-vitamin D might not be sufficient to push macrophages from an inflammatory state to a reparative state, since the M1 macrophages do not seem to respond well to 25-hydroxy-vitamin D stimulus. Therefore, 1,25-dihydroxy-vitamin D from surrounding cells in the inflammatory response, and other signaling molecules, may be important for switching from inflammatory to reparative macrophages. This could also explain why vitamin D supplementation in general has failed to treat chronic inflammation in randomized studies [21,22]. The supra-physiological concentration of 25-hydroxy-vitamin D (1000 nM) used in this study to stimulate the MDMs is a limitation. This concentration was used to be certain not to miss any 3-epi-25-hydroxy vitamin D induction. It is always difficult to mimic physiological conditions in in vitro experiments, especially with vitamin D. It is difficult to know which concentration the cells “see” in vivo (the total concentration of protein-bound vitamin or the free concentration). However, we cannot be certain that the regulatory mechanisms shown in this study are exactly the same in vivo. Another limitation of this study is that our mRNA data were not confirmed by protein data or gene-silencing experiments. The amount of mRNA at a certain point in time cannot be taken as a measure of protein levels or activity. However, it can be used as an indicator of which genes are being activated in different cells.

## 4. Methods and Materials

**Figure 5:**
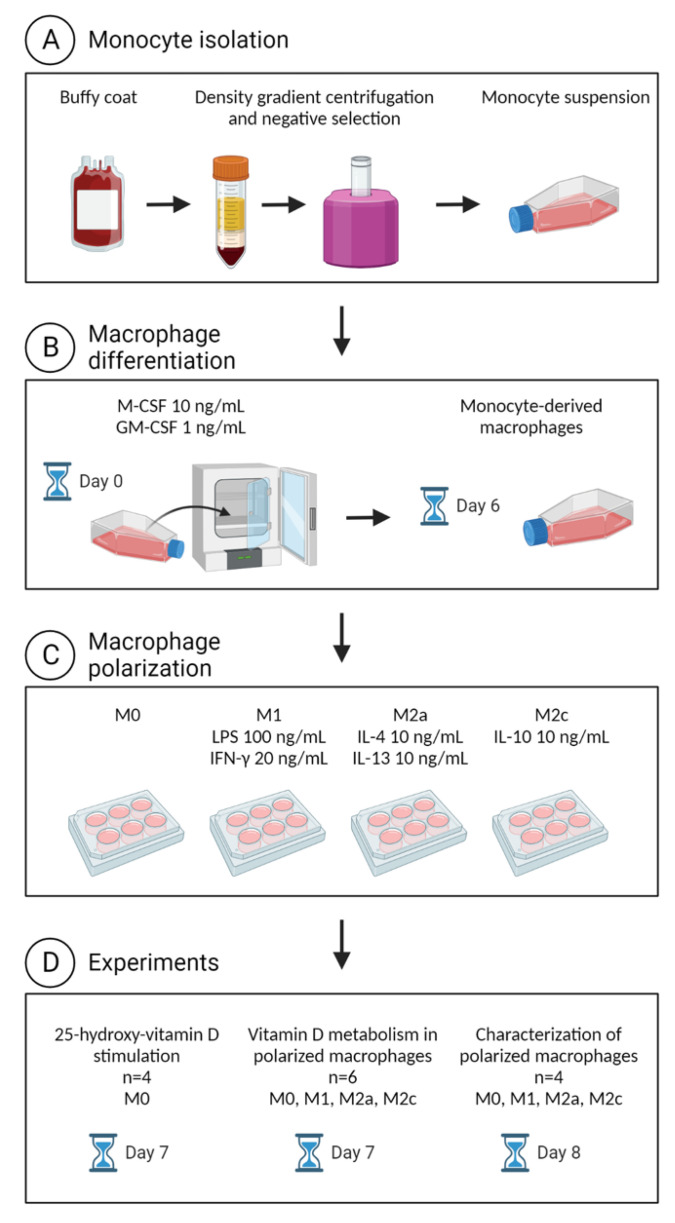
This figure shows the cell isolation and polarization protocol. First, we performed monocyte isolation (**A**), then macrophage differentiation (**B**), then macrophage polarization (**C**) and last the actual experiments (**D**). The figure was created using Biorender.com (accessed on 25 August 2022).

We obtained buffy coats (about 50 mL) from 10 anonymous donors provided by the Department of Clinical Immunology, Aarhus University Hospital, Denmark (Project no. 0094). According to Danish law, the use of anonymized buffy coats does not require specific ethical approval. Monocytes from four donors (D1–D4) were polarized into undifferentiated (M0) MDMs, with the main focus to investigate if 3-epi-25-hydroxy-vitamin D could be detected after stimulation with 25-hydroxy-vitamin D. Monocytes from another six donors (D5–D10) were polarized into M0, M1, M2a and M2c MDMs for the purpose of observing the pattern of vitamin D metabolism in different macrophage subtypes. Figure 5 shows an overview of the cell isolation and polarization protocol.

### 4.1. Monocyte-Derived Macrophages (MDMs)

Buffy coats were diluted 1:1 with 0.9% NaCl and peripheral blood mononuclear cells (PBMCs) were isolated using density centrifugation (400× *g* at RT for 30 min) on a Histopaque^®^-1077 gradient (Sigma Aldrich, Munich, Germany). After isolation, the PBMCs were washed and re-suspended in phosphate-buffered saline (PBS) with 2% fetal calf serum (FCS) (Thermo Fisher Scientific, Waltham, MA, USA) and 1 mM ethylenediamine tetraacetic acid (EDTA) (Merck Millipore, Burlington, MA, USA) and the cell concentration was adjusted to 5 × 10^7^ cells/mL. Monocytes were isolated with negative selection using EasySep™ Human Monocyte Isolation Kit (Stemcell Technologies, Vancouver, BC, Canada) according to the manufacturer’s protocol. The PBMC suspension was incubated with 50 μL/mL isolation cocktail and 50 μL/mL platelet removal cocktail. Magnetic RapidSpheres (50 μL/mL) were added to the cell suspension and after incubation, the tube containing PBMCs was placed in the “Big Easy” (StemCell Technologies) magnet, allowing magnetically labeled non-monocyte cells to attach to the sides of the tube and the monocytes to be poured off. After incubation, the purified monocytes were poured into a tube and centrifuged. The supernatant was discarded, and the pellet was resuspended in 10 mL PBS with 1% FCS and centrifuged. The cells were resuspended in RPMI1640 (ThermoFisher Scientific, Waltham, MA, USA) with 10% FCS. Lastly, the monocytes were counted on NucleoCounter^®^ NC250™ using Solution 18 (ChemoMetic A/S, Allerod, Denmark), and the medium was added to a concentration of 1 × 10^6^ cells/mL. For MDM polarization, the purified monocytes were matured for five days in RPMI1640, 10% FCS and 100 U/100 µg/mL penicillin/streptomycin (ThermoFisher Scientific) with 10 ng/mL macrophage colony-stimulating factor (M-CSF) (Peprotech, Stockholm, Sweden) and 1ng/mL granulocyte-macrophage colony-stimulating factor (GM-CSF) (Peprotech). Media were changed every 2–3 days. At day six, the cells were harvested using a detach buffer and the MDMs were seeded and stimulated with either lipopolysaccharide (LPS) (100 ng/mL) and interferon-gamma (IFN-γ) (20 ng/mL) for M1 polarization, IL-4 (10 ng/mL) and IL-13 (10 ng/mL) for M2a polarization, IL-10 (10 ng/mL) for M2c polarization or left untreated for M0 polarization, for 24 h. This protocol for macrophage isolation and polarization is routinely used in our laboratory. We have previously published data on surface markers and informative biomarkers in these cells [23].

### 4.2. Flow Cytometry

Flow cytometry measures were carried out on separate cells in order to describe the surface markers and cell phenotypes following this protocol. Cells were treated as described in the previous section and following 48 h of polarization, the MDMs were harvested, washed in PBS with 0.5% BSA, and resuspended in stain buffer (PBS with 0.5% BSA and 0.09% NaN_3_) at approximately 1 × 10^7^ cells/mL. For each sample, 250,000 cells (25 µL) were used, and staining was performed in a final volume of 34.5 µL. Unspecific antibody binding was blocked by incubation for 15 min at 4 °C with 100 µg/mL human IgG (Beriglobin, CSL Behring, King of Prussia, PA, USA). Antibody stains were added at the concentrations listed in Table 2 and incubated for 30 min at 4 °C. The antibody panel was based on an established panel at our laboratory and includes various markers; however, only CD80, CD206 and CD163 were analyzed for the purposes of this study. Live/dead fixable dye near IF (Thermo Fisher Scientific) was included in all samples to allow for gating on viable cells. Following staining, cells were washed in stain buffer, fixed in PBS with 0.9% formaldehyde and data were acquired using a CytoFLEX S Flow Cytometer (Beckman Coulter, Brea, CA, USA) with CytExpert software version 2.1 (Beckman Coulter). Compensation was performed using single-stained beads (ArC™ Amine Reactive Compensation Bead Kit from Thermo Fisher Scientific #A10346, and BD™ CompBead Plus from Becton Dickinson #560497), and data were analyzed in FlowJo software version 10.8.1 (Becton Dickinson, Franklin Lakes, NJ, USA). The full gating strategy used is presented in Supplementary Figure S1.

### 4.3. Stimulation of MDMs with 25-Hydroxy-Vitamin D

After polarization into M0, M1, M2a and M2c MDMs, the cells were stimulated with 25-hydroxy-vitamin D. Before stimulation, the media was removed, and cells were washed carefully with 10 mL PBS. 25-hydroxy-vitamin D (Cayman Chemical. Item no: 9000683. Mw 400.6) was added in a concentration of 0, 100, 1000 or 10,000 nanomolar (nM) (see Table 1). All vitamin D stimulations were performed in RPMI1640 with 10% charcoal stripped FCS (Thermo Fisher). After 5 min, 1 mL of the media was removed, centrifuged and stored at −80 °C until further analysis. Cells and media were placed in an incubator (5% CO_2_, 37 °C) for 24 or 48 h. After incubation, the media was removed and centrifuged, and the supernatant was stored at −80 °C until further analysis.

### 4.4. Liquid Chromatography-Mass Spectrometry-Mass Spectrometry (LCMSMS) Measurement of Vitamin D Metabolites

For analyses of 25-hydroxy-vitamin D3 and 3-epi-25-hydroxy-vitamin D3, 100 µL of cell media and 300 µL of acetonitrile containing stable isotope-labeled internal standards were added to an Impact protein precipitation plate (Phenomenex, Torrance, CA, USA). After five minutes of orbital mixing, the supernatant was pulled into a collection plate using a vacuum manifold. The collection plate was then heat sealed and placed for analysis on an LCMSMS system consisting of a Nexera (Shimadzu, Kyoto, Japan) HPLC system in front of a 6500 QTRAP mass spectrometer (Sciex, Framingham, MA, USA). The compounds were separated by a 0.1% formic acid/methanol gradient on a Kinetex F5 column (Phenomenex). The mass spectrometer was operated in positive mode using Atmospheric Pressure Chemical Ionization (APCI) and the analytes were monitored via the transitions: *m*/*z* 383.3 -> 257.2 for 25-hydroxy-vitamin D3 and 3-epi-25-hydroxy-vitamin D3, and *m*/*z* 389.3 -> 263.6 for the internal standard. The run time was six minutes, and the limits of detection were 4 nM for both compounds.

For analyses of 1,25-dihydroxy-vitamin D3 and 24,25-dihydroxy-vitamin D3, 150 µL of cell media and 500 µL of acetonitrile containing stable isotope-labeled internal standards were added to a protein precipitation plate, mixed and the supernatant was transferred to a collection plate as described above. The supernatant was then evaporated to dryness by heated nitrogen and after addition of 4-Phenyl-1,2,4-triazoline-3,5-dione (PTAD) in acetonitrile, 10 min of shaking and addition of water, the plate was sealed and placed on the same LCMSMS system as described above for analysis. For practical reasons, the two compounds were analyzed using two separate methods but sharing the exact same setup. The samples were chromatographed on a Kinetex XB-C18 column (Phenomenex) using 0.1% methylamine and methanol as mobile phases. The mass spectrometer was operated in positive mode using electrospray ionization (ESI), and the analytes were monitored via transitions:

- *m*/*z* 623.3 -> 314.2 for 1,25-dihydroxyvitamin D3, and *m*/*z* 629.3 -> 314.2 for the internal standard. Run-time was four minutes, and the limit of detection was 25 M.
- *m*/*z* 623.3 -> 298.2 for 24,25-dihydroxyvitamin D3, and *m*/*z* 629.3 -> 298.2 for the internal standard. Run-time was four minutes, and the limit of detection was 100 nM.

### 4.5. RNA Isolation and cDNA Preparation for qPCR

Cells used for qPCR analysis were lysed in RLT buffer (Qiagen, Sollentula, Sweden) and mixed with 70% ethanol. Cell samples were then transferred to a QIAamp spin column (Qiagen) centrifuged and washed (RW1 buffer (Qiagen)), then treated with DNase (15 min, room temperature), washed and centrifuged. Samples were resuspended in H_2_O and the RNA concentration was measured by NanoDrop 2000 spectrophotometer (Thermo Fisher Scientific, Waltham, MA, USA). cDNA was synthesized with the High-capacity cDNA reverse transcription kit (Thermo Fisher Scientific) according to the supplier’s recommendations. For the generation of cDNA, 0.1 µg of RNA was used in a total reaction volume of 10 µL using random hexamer priming.

### 4.6. qPCR Analysis

qPCR was conducted on a Lightcycler 480 II PCR instrument (Roche, Basel, Switzerland) with Sybr Green for quantification. The reaction took place in a total volume of 10 µL of the 480 Sybr Green Master Mix Buffer, 250 nM of each primer (Eurofins Genomics, Luxembourg) and 1 µL cDNA prepared as described above. A standard curve was generated for each qPCR reaction based on serial dilutions of the sample with the highest amount of the specific target. The mRNA concentrations were calculated from the standard curve. All samples were run in duplicate, and the mean value was used for analysis.

The primer sequences and annealing temperatures are presented in Table 3. The primer sequences for *CYP27B1* and *CYP24A1* are from the literature [7,24]. The correct identity of all PCR products was confirmed by sequencing.

The household gene *YWHAZ* was found to be constantly expressed in all groups, and target genes are therefore presented relatively to the expression of this gene in the results.

### 4.7. Statistics

Groups (M0, M1, M2a and M2c) were compared using a one-way ANOVA. For flow cytometry data, a one-way ANOVA with repeated measures was used. When a significant effect of groups was found, individual groups were compared using Tukey’s multiple comparisons test. A *p*-value < 0.05 was considered significant. Statistic tests and graphs were performed using Graph Pad Prism version 9 (GraphPad Software, San Diego, CA, USA).

## 5. Conclusions

In conclusion, the metabolism of vitamin D in human MDMs was found to be highly dependent on macrophage polarization. When MDMs are polarized in a reparative direction, the cells have a continuous mRNA expression of *CYP27B1* for synthesizing active vitamin D, and they have decreased vitamin D degradation together, resulting in high 1,25-dihydroxy-vitamin D secretion. When the MDMs are polarized in an inflammatory direction, the cells produced a small amount of active vitamin D, secreted almost no 3-epi-25-hydroxy-vitamin D and upregulated the non-functioning mRNA splice variant for the main degrading enzyme (*CYP24A1-SV*), together resulting in almost no metabolism of the added 25-hydroxy-vitamin D. Furthermore, we showed that human reparative, but not inflammatory, MDMs are able to synthesize 3-epi-25-hydroxy-vitamin D. We believe that these findings have many interesting perspectives on how vitamin D can affect the balance of macrophage polarization and the other way around.

## Figures and Tables

**Table 1:** Concentrations of 3-epi-25-hydroxy-vitamin D 24 h after stimulation with 25-hydroxy-vitamin D in media from M0 MDMs.

25-Hydroxy-Vitamin D Stimulation	Donor 1	Donor 2	Donor 3	Donor 4
0 nM	<4	<4	<4	<4
100 nM	<4	No data	No data	No data
1000 nM	39	No data	37	43
10,000 nM	353	<4	547	489

Donor 1–4 represent four different cell studies with human MDMs. The cells were stimulated with the given concentrations of 25-hydroxy-vitamin D for 24 h and concentrations of 3-epi-25-hydroxy-vitamin D were afterwards measured in the media with LCMSMS. Our limit of detection was 4 nM. All concentrations are in nM.

**Table 2:** Antibodies Used in Flow Cytometry Panel.

Specificity	Fluorophore	Manufacturer	Clone	Catalog No.	Concentration
CD80	Brilliant Violet 510	BD Biosciences, Minneapolis, MN, USA	L307.4	563084	0.906 µg/mL
CD206	Alexa Fluor 700	BioLegend, San Diego, CA, USA	15-2	321132	1.359 µg/mL
CD163	PE	BioLegend, San Diego, CA, USA	GHI/61	333606	1.812 µg/mL

**Table 3:** Primers for qPCR.

	Forward Primer	Reverse Primer	Annealing Temp.
***CYP27B1***	TTGCTATTGGCGGGAGTGG	TGCCGGGAGAGCTCATACAG	60
***CYP24A1***	AGGGGGTCTCAAGAAACAGC	GGCCTTCCACGGTTTGATCT	60
***CYP24A1 SV***	GGACACCTCAAAATCCCTGAACCCAA	CCATAAAATCGGCCAAGACCTCATTG	60
***YWHAZ***	ACTTTTGGTACATTGTGGCTTCAA	CCGCCAGGACAAACCAGT	59

## Data Availability

Not applicable.

## Supplementary Materials

The following supporting information can be downloaded at: https://www.mdpi.com/article/10.3390/ijms231810943/s1.
