# Supplementary figures and images for: Metabolism of 25-Hydroxy-Vitamin D in Human Macrophages Is Highly Dependent on Macrophage Polarization
Source: Int J Mol Sci. 2022 Sep 19;23(18):10943. doi: 10.3390/ijms231810943 (PMC9505540; doi:10.3390/ijms231810943)

## Slide 1
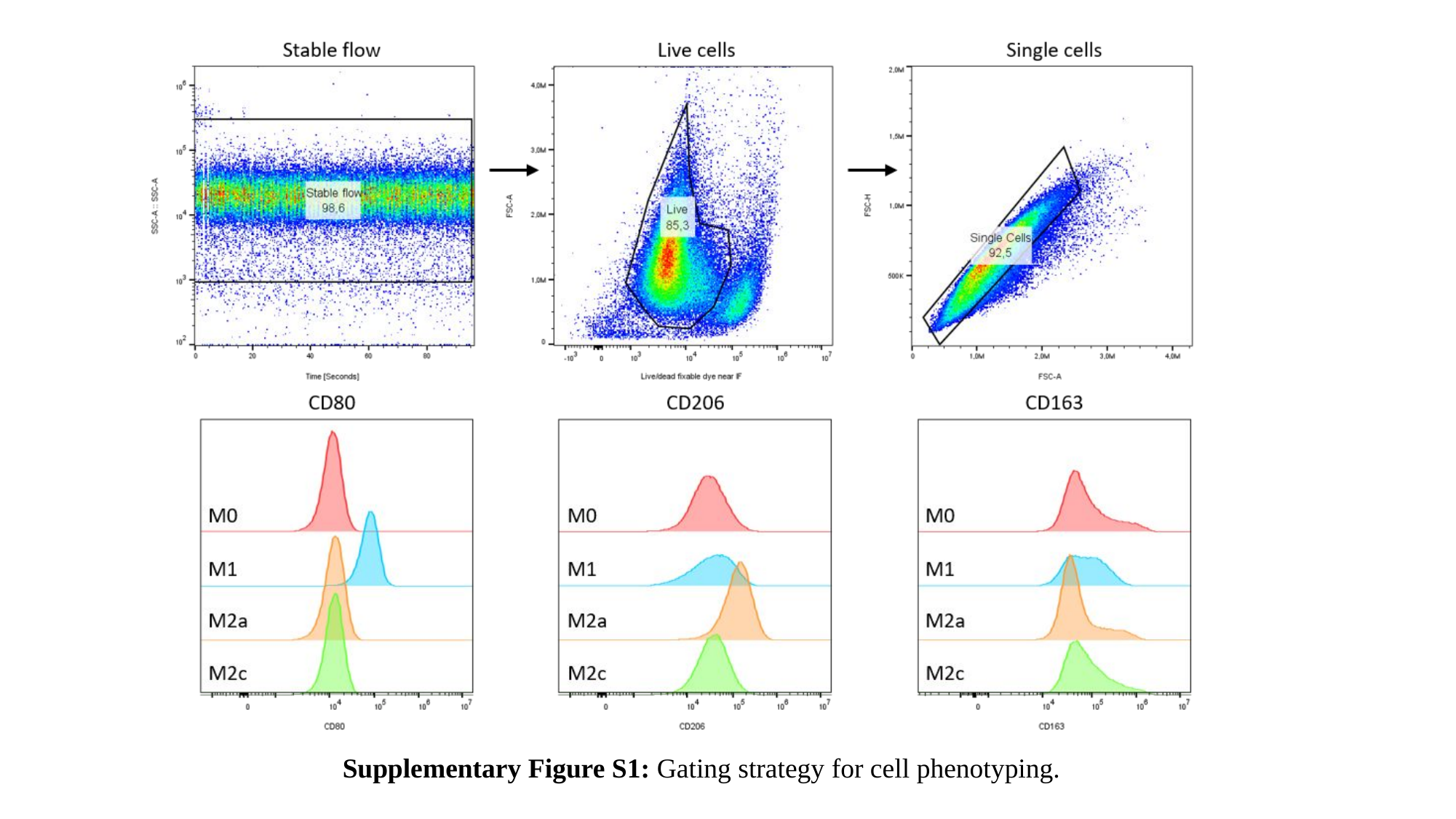

Supplementary Figure S1: Gating strategy for cell phenotyping.

Supplement: Supplementary file 1 [file ijms-23-10943-s001.zip › ijms-1840806-supplementary.pptx]
